# Glucose dynamics during ultramarathon running: a multi-phase framework and implications for late-stage glucose elevations

**DOI:** 10.3389/fspor.2026.1843846

**Published:** 2026-05-28

**Authors:** Kengo Ishihara, Hiromi Kosaka, Hirokazu Taniguchi, Hiromi Kakizaki

**Affiliations:** 1 Faculty of Agriculture, Ryukoku University, Shiga, Japan; 2 Research Center for Functions of Fermented Food, Ryukoku University, Shiga, Japan

**Keywords:** carbohydrate intake, continuous glucose monitors, glucose dynamics, glucose variability, inflammation, insulin sensitivity, sports nutrition, ultramarathon

## Abstract

Ultramarathon running is characterized by extreme duration and substantial physiological stress, making individualized nutritional strategies essential for performance and completion. This mini-review examines glucose dynamics during ultramarathon running using continuous glucose monitors (CGMs) and proposes a conceptual multi-phase framework. Glucose dynamics during ultramarathon running can be conceptualized into three phases. The first phase is characterized by transient elevations in blood glucose. In the second phase, a gradual decline in blood glucose is observed. The third phase is characterized by an increase in glucose variability, leading to transient elevations in blood glucose. Importantly, late-stage increases in blood glucose levels cannot be fully explained by conventional determinants such as carbohydrate intake or exercise intensity. Potential mechanisms underlying these observations include changes in substrate utilization and cumulative inflammatory stress. These changes may impair glucose uptake in skeletal muscle and contribute to a mismatch between glucose availability and utilization. Although direct evidence remains limited, CGMs may provide a useful tool for identifying overall trends in glucose dynamics and supporting individualized nutritional strategies during ultramarathon running.

## 1 Introduction

In recent years, there has been a global increase in the number of runners participating in ultramarathon. Depending on the topography, elite athletes may finish in approximately 18 h, while slower participants may take up to 50 h. Due to the extreme duration of these events, nutritional strategies during competition are critically important.

Ultra-endurance events are defined as sporting activities lasting more than 6 h (51). The nutritional strategies for ultra-endurance athletes remain scientifically underexplored, and are not adequately addressed by existing carbohydrate intake guidelines for endurance exercise (6). In endurance events lasting less than 6 h, higher carbohydrate intake is generally associated with the maintenance of endurance performance. During up to 5 h of cycling, exogenous carbohydrate oxidation rates can reach 1.4 g/min when glucose and fructose are ingested in combination, supporting an intake recommendation of 100 g/h (25). Similarly, studies examining the effects of carbohydrate intake at 60 g, 90 g, and 120 g per hour during a 4.5-hour hilly run found that the 120 g/h group exhibited better maintenance of muscle strength, aerobic capacity, and lower liver and muscle damage markers compared to the lower carbohydrate intake groups (46, 49).

The International Society of Sports Nutrition's position stand on trail running targets elite athletes and recommends carbohydrate intake of 30–50 g/h and protein intake of 5–10 g/h (45). These values are lower than the 90 g/h recommendation of the International Olympic Committee (IOC) for endurance athletes (6) but are substantial for most of the amateur runners. Even in the 24-hour World Championships, the average carbohydrate intake was 63 g/h, with individual values ranging from 13 to 105 g/h, indicating a nearly tenfold difference (32). In ultramarathons, more than 50% of participants reportedly fail to consume more than 30 g/h of carbohydrates (28, 33). These results suggest that the recommended amount of carbohydrate intake is difficult to achieve for all ultramarathon runners. Costa et al. suggested that carbohydrate intake should be tailored to each individual's capacity and comfort for ultramarathon running (9).

Continuous glucose monitors (CGMs) were originally developed for the management of diabetes, and are used to measure interstitial fluid glucose levels, which exhibit a strong correlation with blood glucose levels (40, 44). Although one of the known limitations of CGMs is the occurrence of measurement delays, estimated to be between 5 and 10 min (31, 50), the use of CGMs has enabled endurance athletes to adjust their carbohydrate intake based on nearly real-time changes in interstitial fluid glucose levels (3, 5, 15, 20). However, CGMs-derived glucose values represent only a limited aspect of overall carbohydrate availability. In particular, CGMs do not directly reflect muscle glycogen content or glucose flux, and therefore its physiological interpretation requires caution for endurance athletes (13, 19, 48). CGMs may be considered a potential candidate for the guidance of nutritional strategies for endurance athletes. However, current evidence does not support a clear causal relationship between monitoring glucose levels and endurance performance.

Our recent observation suggests the possibility of increased fluctuations in glucose responses during ultramarathon running (21). Such findings point to the existence of phase-specific glucose dynamics that have not been fully characterized. Therefore, the purpose of this mini-review is to summarize current evidence on glucose dynamics during ultramarathon running. It will propose a hypothesis-driven conceptual framework describing these responses as a multi-phase pattern.

## 2 Glucose dynamics during ultramarathon running: a conceptual multi-phase framework

**Figure 1:**
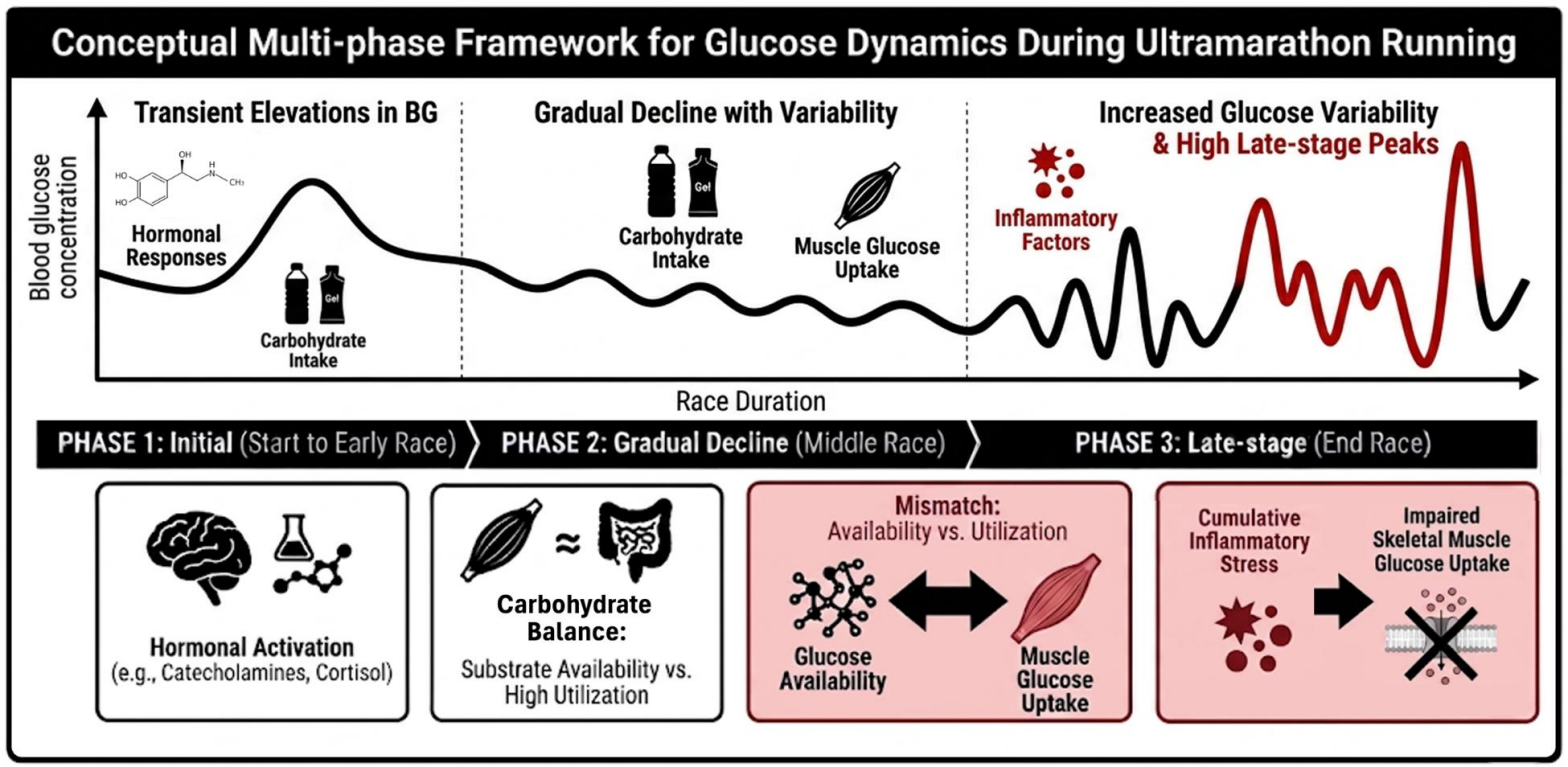
Schematic representation of a three-phase model of blood glucose dynamics during a 160-km ultramarathon. In the first phase, an exercise-associated increase in blood glucose levels is observed. However, when carbohydrates are ingested before the start and blood glucose levels remain elevated, a transient decline may occur at exercise onset. In the second phase, blood glucose levels gradually decline, reflecting an inability to maintain glucose levels during prolonged exercise. In the third phase, even small amounts of carbohydrate intake may result in pronounced elevations in blood glucose levels, accompanied by increased glycemic variability and an overall upward trend. The schematic is based on data from previous work by the authors (21). BG; blood glucose.

Blood glucose regulation during ultramarathon running is not constant and reflects the combined effects of substrate availability, hormonal responses, and cumulative physiological stress (27). While traditional perspectives have emphasized the risk of hypoglycemia during prolonged endurance exercise (14), recent advances in CGMs have revealed the existence of more complex glucose fluctuations in field conditions (37, 43). Despite the presence of individual variability, glucose dynamics during ultramarathon running may be conceptualized based on observations from several studies. In this review, these dynamics are described as a three-phase pattern, as illustrated in Figure 1.

In the first phase of exercise, blood glucose levels may transiently increase compared with resting conditions. The response has been observed across various forms of physical activity and is not specific to ultramarathon running (21, 37, 42, 43), and has also been observed in professional cyclists (17, 53) In some cases, carbohydrate consumption prior to the exercise results in a transient decline in blood glucose levels during the initial phase of exercise. This phenomenon may be interpreted as a combination of the effects of insulin and exercise on lowering blood glucose levels (29, 52).

In the second phase, blood glucose levels tend to decline. As noted above, it has been reported that in excess of 50% of runners do not consume a sufficient amount of carbohydrate (28, 33). Blood glucose levels may gradually decrease during this phase due to insufficient carbohydrate intake. In non-elite trained runners, our study has reported that non-finishers of ultramarathon exhibit a decrease in interstitial fluid glucose levels prior to their immediate dropout (21). Avoiding hypoglycemia during this second phase may be considered as a nutritional target in ultramarathon nutrition, while hypoglycemia is observed in only a few percent of ultramarathon races (37). There is currently insufficient evidence to demonstrate that CGMs are essential for nutritional strategies among elite runners (19). Thus, the results suggest that the decline in glucose levels may not be observed in all individuals during the second phase.

In the third phase of ultramarathon running, glucose responses may become increasingly variable, and in some cases, transient elevations in blood glucose levels may be observed. A case report of a male runner participating in a 24-hour flat-course event described a shift in glucose dynamics, with increases in blood glucose levels and greater variability observed only after approximately 18 h, but not in the earlier phase of the race (43). Previous observational studies reported that higher glucose levels and increased glycemic variability have been observed in the second half compared with the first half, together with progressive increases in peak glucose levels during ultramarathon running (10, 21, 23, 37, 43). However, these observations are limited, and the extent to which such patterns occur more broadly remains unclear. These observations suggest that glucose responses during ultramarathon running may become increasingly complex during the later stages.

## 3 Potential mechanisms underlying glucose variability

Primary contributors to glucose variability are carbohydrate intake and exercise intensity. During ultramarathon races, athletes consume carbohydrates intermittently and in varying amounts depending on race conditions and individual strategies (32, 35, 39). Increases in blood glucose levels are readily observed following carbohydrate ingestion, particularly when larger amounts are consumed at aid stations (37). High-intensity exercise increases circulating catecholamines, cortisol, and glucagon levels (12, 38). These hormones stimulate hepatic glucose production, thereby increasing and maintaining blood glucose levels during exercises (16, 19, 37).

Changes in substrate utilization may further influence glucose dynamics. Prolonged exercise induces a shift in substrate utilization, whereby glucose is substituted with lipids (1). It may also reduce glucose utilization in skeletal muscle, while alternative utilization of lipids increases during the later stages of endurance exercise. It is well documented that elevated concentrations of circulating free fatty acids are observed during periods of prolonged exercise. (41). An increase in circulating free fatty acids has been suggested to result in impaired insulin signaling pathways (30). However, the effects of free fatty acids on glucose dynamics during ultramarathon running remain to be elucidated.

Inflammatory responses play a key role in promoting the development of hyperglycemia by inducing insulin resistance (8, 47). Prolonged endurance exercise is associated with increases in markers of inflammatory cytokines (7). Muscle damage and inflammatory stress may contribute to late-race metabolic instability, as pace decline and ultramarathon distance have been associated with increases in muscle damage and metabolic stress markers (11, 26). Among athletes participating in ultramarathon events, markers of muscle damage and inflammation, such as creatine kinase, lactate dehydrogenase, and C-reactive protein, have been shown to increase progressively during the race (2, 4, 37). A recent study reported that higher glucose variability, as measured by CGMs, was associated with lower monocyte and basophil counts in participants who had completed a 250-km swim and running race (18). There is a possibility that glucose dysregulation due to an increase in muscle damage and inflammation may be related to a decline in immune response during ultra-endurance exercise. The previous studies suggest the association between exercise-induced inflammation and hyperglycemia. However, it remains unclear whether the coordination of inflammation and alterations in glucose levels occur during the later stages of ultramarathon running.

These previous perspectives suggest that glucose variability during ultramarathon running reflects a dynamic balance between glucose supply and utilization. This balance may be associated with increased utilization of lipids and inflammatory responses.

## 4 Practical implications and future directions

Although direct evidence remains limited, a greater rise in blood glucose levels following carbohydrate intake during the later stages of an ultramarathon, compared with the earlier stages (10, 21, 23, 37, 43). These results suggest a potential reduction in glucose tolerance during ultramarathon race. Such responses may reflect impaired glucose uptake in skeletal muscle, potentially associated with underlying factors such as inflammation. This interpretation suggests that the capacity to utilize ingested carbohydrates may be attenuated under these conditions.

Intestinal absorption is typically considered the primary rate-limiting step for carbohydrate utilization in elite athletes (24). The circulating glucose pool is relatively small (approximately 4 g); therefore, the ingestion of large amounts of rapidly absorbable carbohydrates within a short period may lead to transient elevations in blood glucose levels. In addition, our case reports indicated that even small amounts of carbohydrate intake resulted in marked increases in blood glucose levels during ultra-endurance running (22, 34). These results suggested the possibility of an association between a decrease in peripheral glucose uptake and an increase in blood glucose levels during ultra-endurance races. In such cases, nutritional strategies may need to account for this shift in metabolic limitation.

A greater within-individual range of blood glucose levels (i.e., the difference between peak and lowest values) has been associated with a slower running pace (21, 23, 36). It has been proposed that even if absolute blood glucose levels remain within the normoglycemic range (e.g., from 8 mmol/L to 4.3 mmol/L), greater fluctuations in glucose concentrations may be associated with symptoms of hypoglycemia (19). These observations suggest that nutritional strategies during ultramarathon running may benefit from approaches that minimize rapid fluctuations in blood glucose levels.

Further studies are required to determine whether the elevation in blood glucose levels during the later stages of ultramarathons is associated with impaired glucose tolerance. From a practical perspective, further research is needed to develop training, habitual dietary, and in-race nutritional strategies that aim to prevent impaired glucose regulation during the later stages of ultramarathon running.

## 5 Conclusion

Ultramarathon running may provide a particularly suitable context for the application of CGMs, given its relatively stable exercise intensity, prolonged duration, and the critical importance of nutritional management. However, the extant evidence regarding the effectiveness of CGMs use in both elite athletes and recreational participants remains limited. CGMs have several limitations, including temporal measurement delays and an inability to reflect muscle glycogen levels. On the other hand, as discussed in this review, CGMs have the potential to serve as a useful tool for identifying overall trends in blood glucose fluctuations, assessing the current metabolic state of the runners, and supporting the nutritional strategies.
